# Supplementary material for: A framework to infer de novo exonic variants when parental genotypes are missing enhances association studies of autism
Source: bioRxiv. 2025 Jul 24:2025.07.24.666675. Preprint. [Version 1] doi: 10.1101/2025.07.24.666675 (PMC12330658; doi:10.1101/2025.07.24.666675)
Supplement: 3 [file nihpp2025.07.24.666675v1-supplement-3.pdf]

bioRxiv preprint doi: https://doi.org/10.1101/2025.07.24.666675; this version posted July 24, 2025. The copyright holder for this preprint (which was not certified by peer review) is the author/funder, who has granted bioRxiv a license to display the preprint in perpetuity. It is made available under aCC-BY 4.0 International license.

Supplementary Material A : Method Details

Data preprocessing

De novo data We obtained all de novo and inherited variant data from Fu et al. (2022) for ASC and SPARK, separately. All de novo variants incorporated in this study were used directly as provided. Additional postprocessing filters were applied to inherited variants so that the final dataset was enriched for true associations. Specifically, we added an additional within-family segregation filter: variants were retained only if they were transmitted exclusively to affected children or exclusively to unaffected siblings, but not to both. This approach is based on the assumption that ultra-rare variants transmitted to both affected and unaffected siblings are unlikely to meaningfully contribute to autism risk. To further improve signal quality, we filtered inherited variants to those with allele frequency leq 0.00005 in the non-neuro subset of gnomAD v2.1.1 and leq 0.0001 in their respective internal datasets. Inherited PTVs were only retained if classified as high-confidence (HC) by the LOFTEE plugin for VEP and allowed only the "SINGLE_EXON" flag among LOFTEE annotations. All variants were subsequently annotated with CCR scores (Havrilla et al., 2019), gene-level LOEUF metrics (Karczewski et al., 2020), expected and observed predicted loss-of-function variant counts (exp_lof and obs_lof, respectively), Developmental Delay FDR scores (Fu et al., 2022) and gnomad non-neuro AF, when available. Otherwise, variant frequencies were approximated within the appropriate dataset. Note that CCR coordinates were printed in bedfiles, which begin incrementing at 0. Therefore, we added 1 to all start and end coordinates to ensure compatibility with all VCF files.

Case-Control data We obtained case-control variant data from Fu et al. (2022). The case-control cohort contains 1,370 autism cases (n_{female} = 352, n_{male}=1,018) and 4,249 unaffected controls (n_{female} = 2,023, n_{male} = 2,226). Variants were annotated with allele frequency data from gnomAD v2.1.1; when unavailable, allele frequencies were approximated using internal cohort-specific estimates. Consistent with the approach used for filtering and annotating de novo variation, case-control variants were also filtered using a non-neuro subset of gnomAD v2.1.1 allele frequency threshold of leq 0.001. As with the trio data, all variants were subsequently annotated with CCR scores (Havrilla et al., 2019), gene-level LOEUF metrics (Karczewski et al., 2020), expected and observed predicted loss-of-function variant counts (exp_lof and obs_lof), Developmental Delay FDR scores (Fu et al., 2022), and gnomAD non-neuro allele frequencies, when available.

Details of the classifier training procedure

We use rare variant information from the SPARK family-based dataset, which includes data from 7,008 families. Each variant in this dataset is labeled with its inheritance class and accompanied by six offspring-level covariates that help distinguish between these classes. The fraction of *de novo* variants is approximately 0.0063 times that of inherited variants. In such scenarios, a naive application of a classifier tends to assign most variants to the inherited group—the majority class. While this approach may achieve a reasonable overall accuracy, it is not effective for the actual purpose of classification. This highlights the need for algorithms specifically designed to handle imbalanced data. Such algorithms typically rely on ensembling results from multiple learners, each trained on a subset of samples that are balanced through either oversampling the minority class or undersampling the majority class.

After an initial screening of various ensemble algorithms (results not shown), we selected two methods that use random undersampling of the majority class: RUSBoost (Seiffert et al., 2009) and Underbagging (Barandela et al., 2003). A key difference between these two algorithms is that RUSBoost iteratively selects training samples for each learner, assigning greater weight to samples that were misclassified by previous learners. In contrast, Underbagging builds learners in parallel, each trained on a randomly under-sampled, balanced subset of the data. The outputs of these algorithms are scores ranging from 0 to 1, where a higher score indicates that a variant is more likely to belong to the de novo class. A specific threshold can then be applied to these scores to complete the classification procedure. For example, applying a threshold of 0.7 means that a variant is classified as de novo only if its score exceeds 0.7.

RUSBoost and Underbagging require several tuning parameters. The imbalance ratio parameter refers to the intended imbalance ratio for each learner, defined as the ratio of majority instances to minority instances after class rebalancing. The size parameter specifies the number of learners. Additionally, a choice of algorithm is required to construct the learners. We use the random forest algorithm, which includes a parameter for selecting the number of trees. To select these parameters, we divided the dataset into five equally sized folds. Four folds were used to train the model, and the remaining fold was used for validation. The performance metric was constructed based on two scores: precision and recall, defined as:

$$\text{Precision} = \frac{\text{True positives}}{\text{True positives} + \text{False positives}} \text{ and } \text{Recall} = \frac{\text{True positives}}{\text{True posi tives} + \text{False Negatives}},$$

where we define *de novo* class as positive. Tested parameters include ir=1, 1.5, 2 ; es=10, 20, 30; ntree=20, 50, 100 (where appropriate). Note that the LOEUF composition of variants in each bin was roughly identical by design. The final decision on the tuning parameters was based on the area under the Precision-Recall (PR) curve, which evaluates the performance of binary classification algorithms across different threshold levels (Figure S1). The final AUC value is averaged over the results from five test folds.

The results show that the mean area under the PR curve does not vary greatly across different parameter choices, illustrating that the classifier is robust to the selection of parameters (Table S1). Considering both performance and computational complexity, we selected an imbalance ratio of 2, ensemble size of 30, and number of trees per learner as 20 for both RUSBoost- and Underbagging-based classifiers. The PR curves for this set of parameters are also largely similar between RUSBoost and Underbagging, suggesting that the overall performance of the two algorithms is comparable (Figure S1). In the main analysis, when we tested the trained classifiers on the ASC family-based data, we found that RUSBoost identified fewer *de novo* variants than Underbagging at the same

bioRxiv preprint doi: https://doi.org/10.1101/2025.07.24.666675; this version posted July 24, 2025. The copyright holder for this preprint (which was not certified by peer review) is the author/funder, who has granted bioRxiv a license to display the preprint in perpetuity. It is made available under aCC-BY 4.0 International license.

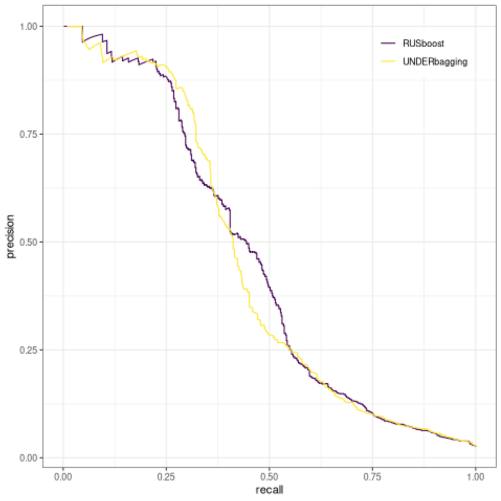

Fig. S1. PR curve for parameters imbalance ratio=2, size=30, ntree=20 (fold 1)

Model	Imbalance ratio	Size	ntree	Mean.PR.AUC
rusboost	1	10	20	0.404
rusboost	1	30	50	0.413
rusboost	1.5	10	20	0.427
rusboost	1.5	20	50	0.437
rusboost	2	20	50	0.451
rusboost	2	30	20	0.454
rusboost	2	30	100	0.453
underbagging	1	10	20	0.411
underbagging	1	30	50	0.426
underbagging	1.5	10	20	0.434
underbagging	1.5	20	50	0.443
underbagging	2	20	50	0.454
underbagging	2	30	20	0.457
underbagging	2	30	100	0.459

Table S1. Mean PR AUC result for parameter selections.

threshold. This difference does not indicate that Underbagging outperforms RUSBoost; rather, it reflects that the same threshold corresponds to a lower point on the recall axis for the RUSBoost-based classifier.

Details of the Random Draw Model

The random draw model first constructs the likelihood of having observed values of likely de novo and likely inherited variants under each risk gene and non-risk gene scenario. It then calculates a Bayes factor, defined as the ratio of this two likelihoods, as the evidence supporting the gene being a risk gene.

Background of the random draw model

The $TADA$ models is a well-established framework to model the number of *de novo* and inherited variants based on genetic parameters under both risk gene and non-risk gene scenarios. Both *de novo* and inherited variants contribute to the evidence supporting the gene being risky. A likelihood ratio, based on the observed number of variants, combined with estimated genetic parameters, provides a risk score for a specific gene. While both *de novo* and inherited variants contribute to the evidence supporting the gene being risky, results from Fu et al. (2022) show that while the gene risk score is positively correlated with the number of *de novo* variants, it is negatively correlated with the number of inherited variants (Figure S2). Most risk genes have a very limited number of inherited variants, while they have a relatively many number of *de novo* variants.

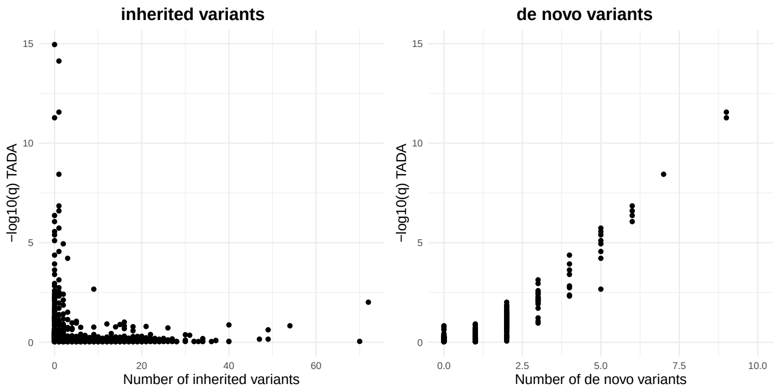

Fig. S2. Marginal relationships between gene risk scores and the number of variants per gene: the number of inherited variants (left) and the number of de novo variants (right). Gene risk scores are represented as -log(q-value) computed in Fu et al. (2022).

The random draw approach models this marginal relationship rather than establishing structural equations for the number of variants. Specifically, it assumes that each variant is a randomly drawn observation from a mixed pool of *de novo* and inherited variants and estimates the likelihood of the observed composition of *de novo* and inherited variants. Generally, the probability of a

bioRxiv preprint doi: https://doi.org/10.1101/2025.07.24.666675; this version posted July 24, 2025. The copyright holder for this preprint (which was not certified by peer review) is the author/funder, who has granted bioRxiv a license to display the preprint in perpetuity. It is made available under aCC-BY 4.0 International license.

random variant being *de novo* is higher for risk genes. Therefore, given a fixed total number of variants, the more *de novo* variants there are, the more likely the gene is to be a risk gene.

With this approach, we can effectively deal with the uncertainty of the inheritance class involved in the case data, as explained in the subsection below. Additionally, even though this approach takes a different perspective to model the observed numbers of variants, it remains highly consistent to the original TADA model when there is no uncertainty regarding the inheritance class (see Figure S3).

Formulation

Let D_i denote the risk status of the gene i, where $D_i = 1$ if the ith gene is a risk gene and $D_i = 0$ otherwise. Let x_{d_i} and x_{h_i} denote the number of likely de novo and likely inherited variants, respectively, oberved in gene i. Also, let l_i^j denote the likely denovo status of the single variant j of gene i; $l_i^j = 1$ if it is likely de novo and $l_i^j = 0$ if it is likely inherited. Then the likelihood of observing x_{d_i}, x_{h_i} under risk and non-risk gene scenarios are formulated as follows:

$$P(x_{h_i}, x_{d_i}|D_i = 1) = \binom{x_{h_i} + x_{d_i}}{x_{d_i}} P(l_i^j = 1|D_i = 1)^{x_{d_i}} (1 - P(l_i^j = 1|D_i = 1))^{x_{h_i}} \quad (1)$$

$$P(x_{h_i}, x_{di}|D_i = 0) = \binom{x_{h_i} + x_{d_i}}{x_{d_i}} P(l_i^j = 1|D_i = 0)^{x_{d_i}} (1 - P(l_i^j = 1|D_i = 0))^{x_h}. \quad (2)$$

The quantities $P(l_i^j = 1|D_i = 1)$ and $P(l_i^j = 1|D_i = 0)$ denotes the probability that randomly selected variant is likely de novo for risk genes and non-risk genes, respectively. Let m_i^j be the indicator of variants being *de novo*, where $m_i^j = 1$ if *de novo* and $m_i^j = 0$ otherwise. Then we have

$$P(l_i^j = 1|D_i = 1) = P(l_i^j = 1|m_i^j = 1, D_i = 1)P(m_i^j = 1|D_i = 1) + P(l_i^j = 1|m_i^j = 0, D_i = 1)P(m_i^j = 0|D_i = 1),$$
$$P(l_i^j = 1|D_i = 0) = P(l_i^j = 1|m_i^j = 1, D_i = 0)P(m_i^j = 1|D_i = 0) + P(l_i^j = 1|m_i^j = 0, D_i = 0)P(m_i^j = 0|D_i = 0).$$

Here we assume that the probability that a *de novo* variant is observed as likely de novo and a inherited variant is observed as likely inherited is indifferent between risk and nonrisk genes, that is :

$$P(l_i^j = 1|m_i^j = 1) = P(l_i^j = 1|m_i^j = 1, D_i = 1) = P(l_i^j = 1|m_i^j = 1, D_i = 1)$$
$$P(l_i^j = 0|m_i^j = 0) = P(l_i^j = 0|m_i^j = 0, D_i = 1) = P(l_i^j = 0|m_i^j = 1, D_i = 0).$$

Then, we can compute the probability that some randomly drawn variant from gene i is likely denovo:

$$P(l_i^j = 1|D_i = 1) = P(l_i^j = 1|m_i^j = 1)P(m_i^j = 1|D_i = 1) + P(l_i^j = 1|m_i^j = 0)P(m_i^j = 0|D_i = 1),$$
$$P(l_i^j = 1|D_i = 0) = P(l_i^j = 1|m_i^j = 1)P(m_i^j = 1|D_i = 0) + P(l_i^j = 1|m_i^j = 0)P(m_i^j = 0|D_i = 0).$$

Two performance parameters $P(l_i^j = 1|m_i^j = 1) := w_1$ and $P(l_i^j = 0|m_i^j = 0) := w_2$ denotes the sensitivity and specificity of the classifier, respectively. They can be estimated empirically in a test sample (ASC family-based data in our application). Also, the quantities $p_i^1 = P(m_i^j = 1|D_i = 1)$ and $p_i^0 = P(m_i^j = 1|D_i = 0)$ are genetic parameters representing the probabilities that a variant drawn from gene i is a de novo variant, under the risk-gene and non-risk-gene scenarios, respectively. The parameter p_i^1 is expected to be higher than p_i^0. For given p_i^0, p_i^1, we have

$$\begin{aligned} &P(l_i^j = 1|D_i = 1, p_i^1) \\ &= P(l_i^j = 1|m_i^j = 1)P(m_i^j = 1|D_i = 1, p_i^1) + P(l_i^j = 1|m_i^j = 0)P(m_i^j = 0|D_i = 1, p_i^1) \\ &= w_1 p_i^1 + (1 - w_2)(1 - p_i^1), \end{aligned}$$

and

$$\begin{aligned} &P(l_i^j = 1|D = 0, p_i^0) \\ &= P(l_i^j = 1|m_i^j = 1)P(m_i^j = 1|D_i = 0, p_i^0) + P(l_i^j = 1|m_i^j = 0)P(m_i^j = 0|D_i = 0, p_i^0) \\ &= w_1 p_i^0 + (1 - w_2)(1 - p_i^0), \end{aligned}$$

In our model, the parameters p_i^0 and p_i^1 follows some nondegenerate distributions under each risk and nonrisk scenarios: $p_i^0 \sim P_{0i}$ and $p_i^1 \sim P_{1i}$. To estimate these distributions, we specify the prior distributions $p_{0i} \sim Beta(\alpha_{0i}, \beta_{0i})$ and $p_{1i} \sim Beta(\alpha_{1i}, \beta_{1i})$ and estimate the gene-specific hyperparameters as follows. We group the genes based on their pre-risk status, which are determined by whether the q-values from the TADA model applied to only family-based data are above or below 0.05 (Fu et al., 2022). Then, for each pre-risk and pre-nonrisk gene sets, we fit a logistic regression model for the *de novo* ratio against log_{10}(mutation rate) for each gene, and use the predicted values to represent the mean values μ_{0i} or μ_{1i} of the distributions P_{0i} or P_{1i}. The variance of P_{0i} or P_{1i} are estimated through a jackknife approach, separately, under the assumption that the variance are identical among genes within risk or nonrisk sets. To calculate such variances, we exclude one gene at a time and compute a mean ratio as the total number of *de novo* variants divided by the total number of variants in each pre-risk/pre-nonrisk group. Let $\bar{x}_{1i}$ be the estimated

bioRxiv preprint doi: https://doi.org/10.1101/2025.07.24.666675; this version posted July 24, 2025. The copyright holder for this preprint (which was not certified by peer review) is the author/funder, who has granted bioRxiv a license to display the preprint in perpetuity. It is made available under aCC-BY 4.0 International license.

mean of the *de novo* ratio calculated for genes in each group excluding the ith gene, and let $\mathcal{I}_{\text{risk}}$ and $\mathcal{I}_{\text{nonrisk}}$ are index sets for the pre-risk and pre-nonrisk genes . Then, the jackknife estimator for the variance is provided by

$$\sigma^2_{0,jack} = \frac{n-1}{n} \sum_{i \in \mathcal{I}_{\text{nonrisk}}} (\bar{x}_{1i} - \frac{1}{n}\sum_{i=1}^{n} \bar{x}_{1i})^2$$

$$\sigma^2_{1,jack} = \frac{n-1}{n} \sum_{i \in \mathcal{I}_{\text{risk}}} (\bar{x}_{1i} - \frac{1}{n}\sum_{i=1}^{n} \bar{x}_{1i})^2.$$

Then we convert gene-specific means and variance using formulas for beta distribution parameters;

$$\alpha_{0i} = \mu_{0i}\left(\frac{\mu_{0i}(1-\mu_{0i})}{\sigma^2_{0,jack}} - 1\right), \beta_{0i} = (1-\mu_{0i})\left(\frac{\mu_{0i}(1-\mu_{0i})}{\sigma^2_{0,jack}} - 1\right)$$

$$\alpha_{1i} = \mu_{1i}\left(\frac{\mu_{1i}(1-\mu_{1i})}{\sigma^2_{1,jack}} - 1\right), \beta_{1i} = (1-\mu_{1i})\left(\frac{\mu_{1i}(1-\mu_{1i})}{\sigma^2_{1,jack}} - 1\right)$$

Finally, the evidence of $D = 1$ against $D = 0$ can be calculated as a Bayes Factor, which accounts for uncertainty in the parameters (p_{0i}, p_{1i}) using gene-specific prior distributions P_{0i} and P_{1i}:

$$BF_{i,RD} = \frac{P(x_h, x_d|D_i = 1)}{P(x_h, x_d|D_i = 0)} = \frac{\int P(x_{h_i}, x_{d_i}|D_i = 1, p_i^1)dP_{1i}}{\int P(x_{h_i}, x_{d_i}|D_i = 0, p_i^0)dP_{0i}}, \tag{3}$$

where the likelihoods are integrated over the distribution of p_i^1 and p_i^0.

Robust check for the random draw model

To further validate the model, we compare the result from the random draw model and the original TADA model. In our main analysis, the random draw model was only applied to a case data, while the evidence from the family-based data is still collected through the family-based component of the $TADA_{CC}$ data. That is, in our main implementation, the only difference between $TADA_{RD}$ and $TADA_{CC}$ model is the treatment of the case-control data part. However, the random draw model can be still applied to a family-based data by simply setting $w_1 = w_2 = 1$. Since the TADA model has been validated in numerous previous studies for its validity and effectiveness, the comparison between the TADA model and the random draw model for the family-based data will serve as a tool to assess the robustness of our random draw model. A high correlation between two different approaches supports the validity of both methods.

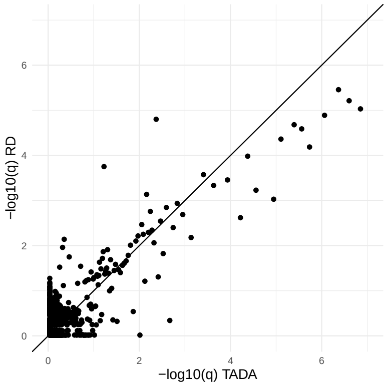

Fig. S3. Comparison of the Random Draw and the TADA model in family-based data.
